# A Potential Urodynamic Classification of Voiding Patterns in Neurogenic Lower Urinary Tract Dysfunction Due to Lower-Level Spinal Cord Injury

**DOI:** 10.3390/jcm15072627

**Published:** 2026-03-30

**Authors:** Shucong Peng, Shan Tian, Xiuming Li, Jin Sun, Ping Chen, Qun Zhang, Xueyan Shen, Jianghong Fu, Junfa Wu, Yulian Zhu, Yi Wu, Gang Liu

**Affiliations:** 1 Department of Rehabilitation Medicine, Huashan Hospital, Fudan University, Shanghai 200040, China; psc0918@163.com (S.P.);; 2 School of Exercise and Health, Shanghai University of Sport, Shanghai 200438, China; 3 Department of Rehabilitation Medicine, Ruijin Hospital, Shanghai Jiaotong University School of Medicine, Shanghai 200025, China

**Keywords:** urodynamic classification, voiding patterns, neurogenic lower urinary tract dysfunction, lower-level spinal cord injury

## Abstract

**Objectives:** To characterize urodynamic findings after lower-level spinal cord injury (LSCI) and to evaluate a new pressure-based classification framework—the bladder–sphincter dyscoordination syndrome (BSDS)—for describing voiding patterns. We also introduce a descriptive “neurogenic bladder outlet obstruction” (NBOO) phenotype for straining-dependent voiding difficulty. **Methods:** We retrospectively analyzed the first urodynamic studies (December 2020–August 2024) in 81 men with LSCI (injury at T10 or below). Key urodynamic measures included detrusor and intravesical pressures during filling and voiding, bladder volumes (first desire to void and capacity), compliance, maximum flow rate (Q_max), post-void residual (PVR), voiding efficiency, and the ratio of detrusor to abdominal pressure rise (ΔPdet/ΔPabd). We compared cases with detrusor overactivity (DO) versus those without DO. Among those with voiding discoordination, we distinguished classical detrusor–sphincter dyssynergia (DSD) from a proposed NBOO phenotype (characterized by abdominal straining pressure ≥ 40 cmH_2_O, detrusor pressure < 20 cmH_2_O, incomplete emptying, and no anatomic obstruction). We further classified discoordination cases using the BSDS framework into four subtypes—dual high-pressure (DHP), detrusor-muscle predominant (DMP), dual low-pressure (DLP), and abdominal-pressure predominant (APP)—based on reference pressure thresholds (detrusor 20 cmH_2_O; abdominal 40 cmH_2_O). **Results:** Patients with DO (43.2%) showed significantly higher detrusor pressures during filling (at first desire to void and at capacity) and a lower first desire volume than non-DO patients, while maximum capacity was similar (*p* = 0.105). During voiding, DO cases had lower PVR and higher emptying rates, although the detrusor-vs-abdominal pressure contribution (ΔPdet/ΔPabd) was comparable between groups. Among 63 patients with voiding discoordination, 32 (50.8%) met NBOO criteria; these NBOO cases exhibited lower detrusor and intravesical voiding pressures but worse emptying (higher PVR) compared to classical DSD cases. Overall, 76 of 81 patients (93.8%) fit within the BSDS classification—distributed as 22 DHP, 13 DMP, 15 DLP, and 26 APP patterns. **Conclusions:** The BSDS framework (and the NBOO descriptor when conventional indices cannot be applied) offers a novel way to describe voiding patterns after LSCI. It links urodynamic observations to potential management strategies (by identifying whether the bladder or outlet is the primary issue). Prospective studies are needed to validate this framework against outcomes such as upper tract integrity, continence, and dependence on catheterization.

## 1. Introduction

Spinal cord injury (SCI) is a devastating neurological condition; approximately 80% of patients develop neurogenic lower urinary tract dysfunction (NLUTD), which compromises quality of life and complicates rehabilitation [1]. After SCI, lower urinary tract dysfunction typically manifests as detrusor overactivity (DO) during storage and impaired or absent volitional voiding during emptying. While DO can often be mitigated pharmacologically, voiding failure remains difficult to correct with noninvasive approaches; medications or electrical/magnetic stimulation frequently yield suboptimal emptying, and many patients ultimately rely on clean intermittent catheterization (CIC) [2,3]. Prolonged catheter dependence carries physical complications (e.g., urethral trauma, infection) and substantial psychosocial and economic burdens, particularly in resource-limited settings [4,5].

Epidemiologically, lower-level spinal cord injury (LSCI; T10 and below, including cauda equina) accounts for about one-quarter of cases [6]. In LSCI, detrusor and urethral sphincter function are impaired, whereas abdominal muscle strength is relatively preserved, producing heterogeneous voiding patterns driven in part by compensatory abdominal straining. Traditional constructs—such as detrusor–sphincter dyssynergia (DSD) or detrusor underactivity (DU)—and pressure–flow indices—such as the bladder outlet obstruction index (BOOI) and bladder contractility index (BCI)—provide only partial explanatory and management guidance in this context, especially when Qmax is near zero or abdominal straining predominates, conditions under which pressure–flow indices are inapplicable or unreliable [7,8,9]. Consequently, there is a need for a clinically practical classification that better describes voiding dysfunction after LSCI and links physiology to management.

To address this gap, we propose an operational classification tailored to LSCI. Bladder–sphincter dyscoordination syndrome (BSDS) is introduced as a broad umbrella encompassing any lack of coordination between bladder pressure generation and sphincter relaxation during voiding. This concept extends beyond the classical definition of detrusor–sphincter dyssynergia (DSD). Notably, ICS defines DSD as an involuntary contraction of the urethral sphincter concurrent with a detrusor contraction, typically seen in supra-sacral injuries [8]. In contrast, lower-level (sacral or cauda equina) injuries seldom exhibit true DSD [8], often showing an atonic or non-relaxing sphincter instead. BSDS encompasses both scenarios—the classical DSD (a high-pressure detrusor contraction against a closed outlet) and other dysfunctional patterns where voiding is ineffective (e.g., absent/weak detrusor contraction with compensatory abdominal straining). We also introduce the term “neurogenic bladder outlet obstruction (NBOO)” as a descriptive label for a functional outlet obstruction due to neurological injury (distinct from mechanical obstruction). Specifically, NBOO here denotes straining-dependent voiding difficulty in LSCI: the detrusor contributes minimal pressure while the abdominal muscles generate high pressure, yet the outlet fails to relax, resulting in poor emptying despite no anatomical blockage. This NBOO construct is intended as a practical descriptor (not a formal diagnosis) and aligns with the ICS concept of a non-relaxing sphincter causing functional obstruction [8]. We stress that BSDS and NBOO are heuristic frameworks to better describe voiding patterns after LSCI—they complement rather than replace standard ICS terms or pressure-flow interpretations.

The conceptual novelty of BSDS lies in mapping the source of voiding force (detrusor vs. abdominal) against outlet behavior (effective vs. obstructed). This yields a matrix of patterns that includes but is not limited to established entities (e.g., DSD or detrusor underactivity). By framing voiding dysfunction in this pressure-based way, we aim to facilitate tailored management (for example, deciding when to target the sphincter vs. boost bladder contractility). However, we recognize these constructs are preliminary and not yet clinically validated. They are meant to generate hypotheses for future research, and their overlap with existing categories (such as ICS-defined DSD or detrusor underactivity) will be examined in our analysis. Potential limitations and misclassifications are discussed later in this report.

Therefore, this study aimed to (i) describe storage- and voiding-phase urodynamic features in men with LSCI, (ii) quantify how often negligible-flow voiding limits the use of conventional pressure–flow indices, and (iii) illustrate a pragmatic BSDS reporting framework, including a straining-dependent NBOO phenotype, as a hypothesis-generating approach to summarize voiding force–resistance interactions in this population.

## 2. Materials and Methods

### 2.1. Study Design and Participants

This retrospective study included consecutive patients with lower-level spinal cord injury (LSCI; T10 and below, including cauda equina syndrome) who were hospitalized in the Department of Rehabilitation Medicine at Huashan Hospital and underwent their first urodynamic study between December 2020 and August 2024. All patients underwent specialized urological examinations or ultrasounds to rule out significant prostatic hyperplasia, and the catheterization process proceeded smoothly to exclude severe urethral strictures.

Inclusion criteria: (i) neurogenic lower urinary tract dysfunction (NLUTD) attributed to LSCI (T10 and below); (ii) clinically stable after spinal shock; (iii) normal urinalysis; (iv) intact cognition, informed consent, and cooperation with testing.

Exclusion criteria: (i) NLUTD due to lesions above T10 or other neurologic etiologies (e.g., stroke); (ii) spinal shock, medical instability, or severe comorbidities precluding testing; (iii) urinary tract infection, gross hematuria, or any contraindication to urodynamics; (iv) structural outlet obstruction sufficient to independently explain abnormal urodynamics, such as clinically significant prostatic enlargement or urethral stricture.

All patients provided written informed consent. The study protocol conformed to the Declaration of Helsinki and was approved by the Huashan Hospital Ethics Committee (approval No. 2020-672).

### 2.2. Urodynamic Protocol

Urodynamic studies followed International Continence Society recommendations [7,10] and comprised uroflowmetry with post-void residual, transurethral cystometry with pressure–flow analysis, and static urethral pressure measurements by a urodynamic analyzer (NDLY-11B; Guangzhou Potent Medical Equipment Joint-Stock Co., Ltd., Guangzhou, China). Bladder filling was performed with normal saline at 60 mL/min in the supine position using an 8 Fr urethral catheter and a 10 Fr balloon rectal catheter. Transducers were zeroed at the level of the pubic symphysis. No surface EMG or video-UDS was available. Filling proceeded until the maximum cystometric capacity or a safety limit of 550 mL, whichever occurred first; filling was temporarily paused for clinically significant events and resumed after stabilization. Studies were interpreted independently by two urodynamics physicians; discrepancies were resolved by consensus.

### 2.3. Operational Definitions and Thresholds [7,8]

Pressures: Intravesical pressure (Pves), abdominal pressure (Pabd), and detrusor pressure (Pdet = Pves − Pabd) were recorded per ICS standards.

Detrusor overactivity (DO): Defined according to ICS as any involuntary detrusor contraction during the filling phase before permission to void.

Compliance: Calculated as ΔVolume/ΔPdet (mL/cmH_2_O). Low compliance was pragmatically defined as <20 mL/cmH_2_O.

Maximum urethral pressure (MUP) and maximum urethral closure pressure (MUCP) were obtained per ICS urethral pressure profilometry standards [10]; static urethral pressure recording was available as described in 2.2.

Voiding indices [10]: Qmax denotes maximum urinary flow. Pdet@Qmax and Pves@Qmax were measured at Qmax when voiding occurred. ΔPdet was defined as Pdet_max − Pdet@CC; ΔPabd as Pabd@max − Pabd@CC.

Rationale for pressure thresholds: We selected a detrusor pressure of 20 cmH_2_O as a pragmatic reference threshold to indicate minimal detrusor contribution. Literature on detrusor contractility suggests that a Pdet@Qmax well below normal (e.g., <20 cmH_2_O) corresponds to severely reduced detrusor strength [11]. In fact, some authors classify voiding contractions with Pdet < 20 cmH_2_O as indicative of severe detrusor underactivity [11]. Thus, Pdet ≥ 20 cmH_2_O was considered a nominal level where active detrusor effort is present, whereas Pdet < 20 implied an almost inactive detrusor. Similarly, we chose an abdominal pressure threshold of 40 cmH_2_O based on clinical safety concerns. Sustained intravesical pressures above ~40 cmH_2_O (whether generated by detrusor or abdominal strain) have historically been associated with risk of upper urinary tract damage [12]. This 40 cmH_2_O threshold, originally noted in pediatric neurogenic bladder studies (e.g., spina bifida [12]), is often used as a warning criterion for unsafe voiding pressures. We therefore used Pabd ≥ 40 cmH_2_O as an indicator of significant straining effort that could endanger bladder or kidney health if chronic. These thresholds were applied to stratify BSDS subtypes but were not treated as rigid cut-offs. Rather, 20/40 cmH_2_O served as reference points to structure our phenotype descriptions and discuss pressure-related risk. All pressure readings were interpreted in the context of the full urodynamic trace, and not in isolation.

### 2.4. BSDS Concept and Subtype Classification

To capture clinically relevant force–resistance interactions, we used Bladder–Sphincter Dyscoordination Syndrome (BSDS) as an operational umbrella for patterns in which intravesical pressure rises (via detrusor contraction and/or abdominal straining) but sphincter relaxation is ineffective, resulting in poor emptying. For descriptive reporting, BSDS was stratified into four reference-threshold subtypes:

① Dual High-Pressure (DHP): Pdet ≥ 20 cmH_2_O and Pabd ≥ 40 cmH_2_O.
② Detrusor-Muscle Predominant (DMP): Pdet ≥ 20 cmH_2_O, Pabd < 40 cmH_2_O.
③ Dual Low-Pressure (DLP): Pdet < 20 cmH_2_O, Pabd < 40 cmH_2_O.
④ Abdominal-Pressure Pattern (APP): Pdet < 20 cmH_2_O, Pabd ≥ 40 cmH_2_O.

As an LSCI-specific operational label—not a replacement for pressure–flow–based obstruction frameworks—“neurogenic bladder outlet obstruction (NBOO)” denoted straining-dependent outlet difficulty after structural obstruction was excluded, with Pabd ≥ 40 cmH_2_O and Pdet < 20 cmH_2_O used as reference thresholds, and inadequate emptying on urodynamics.

In male patients who voided during pressure–flow testing without substantial abdominal straining, BOOI = Pdet@Qmax − 2 × Qmax and BCI = Pdet@Qmax + 5 × Qmax were calculated as exploratory anchors to standard obstruction/contractility indices; these indices were not applied when Qmax ≈ 0 or straining predominated.

Notably, the DMP subtype corresponds to the classic DSD scenario (high detrusor pressure voiding against a contracted sphincter). DLP represents an underactive detrusor pattern (weak or absent detrusor contraction with no significant abdominal straining)—akin to detrusor underactivity in ICS terms. APP denotes voiding almost entirely by Valsalva effort (a functional obstruction scenario with a non-relaxing outlet despite high abdominal push, which we term NBOO). DHP involves both vigorous detrusor contraction and strong straining—a high-pressure pattern that may occur in some cases of DSD with concomitant straining. We emphasize that these labels are descriptive and not diagnostic absolutes; they provide a structured way to discuss the interplay of forces in voiding dysfunction after LSCI.

Because dynamic sphincter assessments (surface EMG/video-UDS) were unavailable, “DSD” in this study refers to a pressure-trace–inferred, DSD-like pattern rather than an EMG-confirmed diagnosis.

### 2.5. Outcomes and Data Handling

Primary descriptive outcomes included filling-phase pressures (at first desire to void and at capacity), CC, compliance, voiding-phase pressures (Pdet@Qmax, Pdet_max, Pves at voiding onset), Qmax, PVR, emptying rate = (voided volume/CC) × 100%, and ΔPdet/ΔPabd. Grouping variables were DO vs. non-DO, DSD vs. NBOO, and BSDS subtypes (DHP/DMP/DLP/APP). Data were extracted from the clinical urodynamic system and curated in Excel; range checks and visual inspection were used to detect entry errors. Missingness was reported; analyses used all available data without imputation.

### 2.6. Statistical Analysis

Analyses were performed in IBM SPSS Statistics for Windows, version 20.0 (IBM Corp., Armonk, NY, USA). Continuous variables are reported as mean ± SD (if approximately normal by Shapiro–Wilk and with homogeneity of variance by Levene), otherwise as median (IQR). Independent-samples *t*-tests or Mann–Whitney U tests compared two groups; one-way ANOVA (with Tukey post hoc) or Kruskal–Wallis (with Dunn post hoc) compared >2 groups, as appropriate. Categorical variables were compared by χ^2^ or Fisher’s exact tests. Two-sided *p* < 0.05 denoted statistical significance.

Given the number of urodynamic variables examined, analyses were considered exploratory/descriptive and were not adjusted for multiplicity; *p*-values should therefore be interpreted cautiously as hypothesis-generating rather than definitive evidence of group differences.

## 3. Results

### 3.1. Basic Information

**Figure 1:**
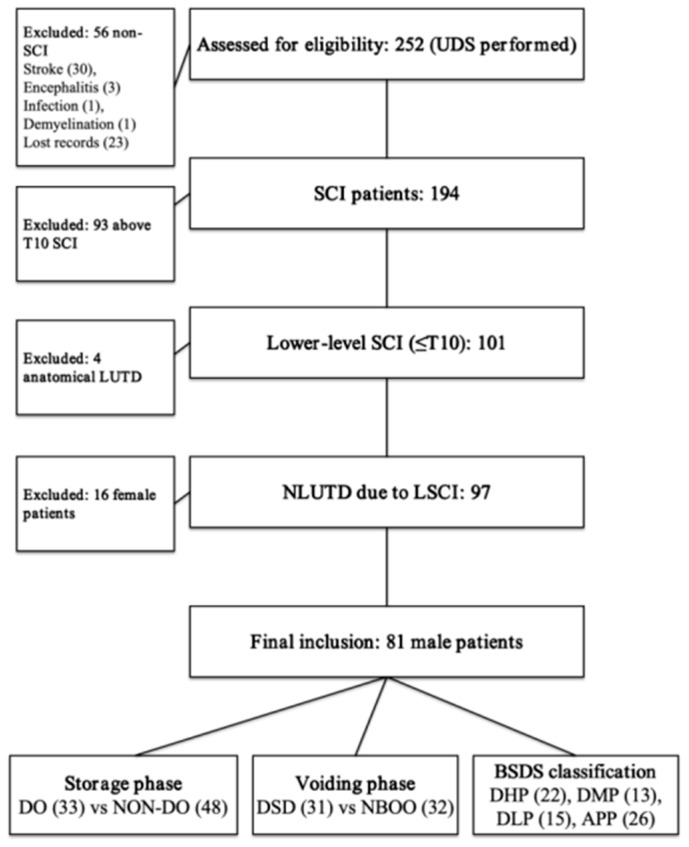
Flowchart of screening, exclusions, and analysis cohort. Note: LSCI: Lower-level Spinal Cord Injury; UDS: Urodynamic Study; DO: Detrusor Overactivity; DSD: Detrusor–Sphincter Dyssynergia; NBOO: Neurogenic Bladder Outlet Obstruction; BSDS: Bladder–Sphincter Dyscoordination Syndrome; DHP: Dual High Pressure; DMP: Detrusor-Muscle Predominant; DLP: Dual Low-Pressure; APP: Abdominal Pressure Pattern.

This retrospective cohort initially included 252 patients with lower-level spinal cord injury who underwent urodynamic testing between December 2020 and August 2024. After applying prespecified exclusions (injury level above T10, active urinary tract infection, incomplete urodynamic records, or female sex), 81 male patients remained for analysis (mean age 40.43 ± 14.18 years; mean time since injury 7.56 ± 6.27 months). The study flow is summarized in Figure 1, and baseline characteristics are provided in Table 1. The male-only analytic cohort was chosen to enable standardized pressure-flow interpretation (including BOOI/BCI in those who voided) and to minimize sex-related heterogeneity in outlet metrics.

### 3.2. Urodynamic Characteristics During the Storage Phase

During cystometry, the bladder was filled with normal saline at a standardized rate (60 mL/min; supine position) until the cystometric capacity (CC) was reached or a safety limit of 550 mL, whichever occurred first. Filling was temporarily paused for clinically significant events (e.g., discomfort, sustained detrusor pressure elevation, leakage) and resumed after stabilization [8]. Detrusor overactivity (DO) was defined according to the International Continence Society as any involuntary detrusor contraction during filling9. Patients without DO were categorized as non-DO. Among the 81 men, 33 exhibited DO (terminal *n* = 12; phasic *n* = 21), and 48 were non-DO. Table 2 summarizes urodynamic parameters for both groups. Significant between-group differences were observed primarily in storage- and voiding-phase measures, whereas static urethral pressure showed no statistical difference.

Compared with the non-DO group, the DO group showed higher detrusor and intravesical pressures at first desire to void (FD) (Pdet 19.13 ± 22.28 vs. 4.99 ± 3.47 cmH_2_O, *p* < 0.001; Pves 25.22 ± 22.64 vs. 9.29 ± 4.78 cmH_2_O, *p* = 0.001). Consistently, the FD volume was lower in the DO group (204.37 ± 127.42 vs. 265.30 ± 120.40 mL, *p* = 0.035). At maximum cystometric capacity (CC), pressures also remained higher with DO (Pdet 34.28 ± 22.80 vs. 9.58 ± 6.04 cmH_2_O, *p* < 0.001; Pves 45.36 ± 28.41 vs. 17.03 ± 8.25 cmH_2_O, *p* < 0.001), whereas CC itself did not differ (451.33 ± 120.38 vs. 496.86 ± 99.56 mL, *p* = 0.105). In line with these pressure differences, compliance was lower in the DO group (12.58 ± 8.30 vs. 53.46 ± 29.28 mL/cmH_2_O, *p* < 0.001), and a larger proportion met the low-compliance criterion of <20 mL/cmH_2_O.

Although DO is fundamentally a storage-phase phenomenon, several voiding-phase parameters differed. The DO group had lower PVR (359.84 ± 163.38 vs. 464.25 ± 163.22 mL, *p* < 0.001), a higher emptying rate (23.13% vs. 10.73%, *p* = 0.021), and higher Qmax (5.16 ± 9.10 vs. 2.39 ± 5.30 mL/s, *p* = 0.031). Pves at voiding onset (Pves@VB) and maximum detrusor pressure (Pdet_max) were also higher with DO (Pves@VB 46.21 ± 30.15 vs. 20.35 ± 14.91 cmH_2_O, *p* < 0.001; Pdet_max 58.53 ± 27.17 vs. 30.82 ± 32.03 cmH_2_O, *p* < 0.001). Pdet@Qmax was significantly higher (34.19 ± 16.84 vs. 20.64 ± 20.60 cmH_2_O, *p* = 0.006), whereas Pves@Qmax did not differ (54.99 ± 27.68 vs. 54.47 ± 43.75 cmH_2_O, *p* = 0.375). Importantly, ΔPdet and ΔPabd did not differ between groups (*p* = 0.188 and *p* = 0.148, respectively).

The lower residuals and higher emptying rate observed in the DO group likely reflect carry-over of involuntary detrusor contractions from the storage phase rather than superior volitional contractility. Given the absence of group differences in ΔPdet/ΔPabd, and without pressure–flow indices of contractility/obstruction in all patients, we refrain from inferring a stronger intrinsic emptying ability in the DO group. Group differences in PVR/emptying rate with DO likely reflect carry-over of involuntary detrusor contractions rather than superior volitional contractility, as ΔPdet/ΔPabd were similar.

### 3.3. Bladder-Sphincter Dyscoordination Syndrome (BSDS) and Its Classification

Micturition is governed by the balance between force (generated by the detrusor and, in some patients, by compensatory abdominal straining) and outlet resistance (primarily urethral sphincter relaxation failure in the absence of fixed anatomic obstruction) [2,3]. Detrusor-sphincter dyssynergia (DSD) denotes a voiding-phase pattern in which the detrusor contracts while the external urethral sphincter fails to relax (or paradoxically contracts), typically associated with suprasacral lesions that disrupt pontine–spinal coordination [9,10]. Classic DSD presupposes a robust detrusor contraction. Because dynamic sphincter assessments (surface EMG/video-UDS) were unavailable, “classic DSD” in this study refers to a pressure-trace–inferred, DSD-suspected pattern rather than an EMG-confirmed diagnosis.

In our LSCI cohort, we observed a sizeable subset of patients who deviated from this classic pattern. These patients voided little or not at all (near-zero Qmax), exhibited weak detrusor activity, and relied predominantly on abdominal straining with Pabd increases ≥ 40 cmH_2_O. In such cases, pressure-flow indices that require a measurable Qmax (e.g., BOOI/BCI) are inapplicable or unreliable—either because Pdet@Qmax does not exist when Qmax = 0, or because substantial abdominal straining confounds the interpretation. During pressure–flow testing, 49/81 (60.5%) men exhibited no measurable volitional flow (Qmax = 0), and 62/81 (76.5%) had minimal flow (Qmax < 5 mL/s). Under these conditions, Pdet@Qmax is absent or confounded, rendering BOOI/BCI inapplicable. Where detrusor-driven, measurable flow occurred without substantial abdominal straining, BOOI/BCI were calculated and used descriptively.

**Figure 2:**
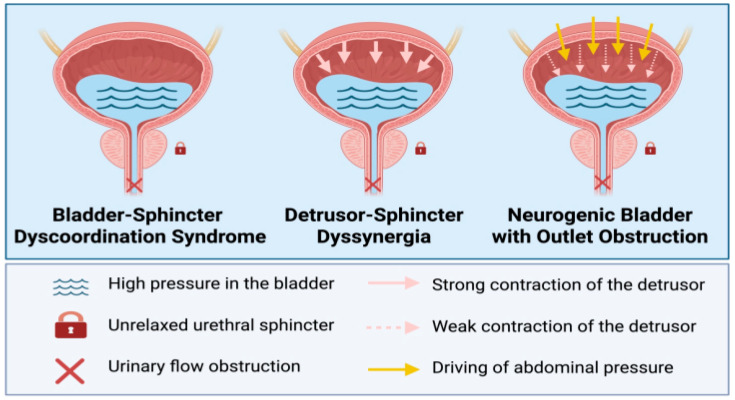
Conceptual schematic of bladder-sphincter dyscoordination syndrome (BSDS), detrusor-sphincter dyssynergia (DSD), and neurogenic bladder outlet obstruction (NBOO).

To facilitate clinical communication in LSCI, we apply a study-specific operational label—neurogenic bladder outlet obstruction (NBOO)—for straining-dependent outlet difficulty after structural obstruction has been excluded, using Pabd ≥ 40 cmH_2_O and Pdet < 20 cmH_2_O as reference thresholds, together with inadequate emptying (see Figure 2). This label does not replace standard, pressure–flow–based obstruction constructs; rather, it highlights a practically important pattern where outlet resistance is not adequately overcome by the available bladder forces.

For clarity, we used a two-step operational approach. First, a pragmatic screening definition (“BSDS criteria”) identified patients with poor emptying and low flow during attempted voiding (Pves > 20 cmH_2_O, Qmax < 15 mL/s, and PVR > 50 mL). Second, BSDS subtypes (DHP/DMP/DLP/APP) were assigned using reference thresholds of Pdet (20 cmH_2_O) and Pabd (40 cmH_2_O). The DSD-suspected vs. NBOO-suspected comparison was performed in the subset with interpretable voiding-attempt traces meeting the discoordination definition (*n* = 63).

Comparative urodynamics (Table 3). Among patients with discoordination (*n* = 63), 32 met the NBOO pattern (50.79%) and 31 had classic DSD (49.21%). Storage phase (at maximum capacity): NBOO exhibited lower pressures—Pdet 16.23 ± 14.99 vs. 29.02 ± 24.39 cmH_2_O (*p* = 0.027) and Pves 24.01 ± 18.83 vs. 39.39 ± 29.02 cmH_2_O (*p* = 0.017)—together with higher compliance (38.77 ± 30.65 vs. 22.97 ± 31.02 mL/cmH_2_O, *p* = 0.009) and a larger FD volume (273.77 ± 135.19 vs. 202.23 ± 124.81 mL, *p* = 0.025). Voiding phase: NBOO showed lower detrusor/intravesical pressures—Pdet@Qmax 21.56 ± 20.39 vs. 33.49 ± 18.49 cmH_2_O (*p* = 0.040), Pves@VB 24.77 ± 16.43 vs. 44.78 ± 31.89 cmH_2_O (*p* = 0.009), and Pdet_max 32.14 ± 22.82 vs. 64.91 ± 33.39 cmH_2_O (*p* < 0.001)—along with a higher PVR (475.76 ± 128.12 vs. 363.69 ± 167.65 mL, *p* = 0.004) and a lower emptying rate (8.46% vs. 20.21%, *p* = 0.029); Qmax tended to be lower (1.46 ± 2.77 vs. 5.29 ± 9.43 mL/s, *p* = 0.083). Δ metrics: NBOO had a lower ΔPdet (15.91 ± 15.87 vs. 35.89 ± 35.68 cmH_2_O, *p* = 0.005) but a higher ΔPabd (81.51 ± 33.38 vs. 61.47 ± 43.45 cmH_2_O, *p* = 0.019). Taken together, these findings indicate a straining-dependent, low-detrusor-force pattern in NBOO, whereas DSD reflects suggestive of outlet non-relaxation (inferred from pressure–flow features) in the presence of a contracting detrusor.

In routine practice—especially outside specialized centers—dynamic sphincter evaluation may be unavailable, and clinicians must infer sphincter behavior from indirect signals. We therefore introduce bladder–sphincter dyscoordination syndrome (BSDS) as a clinical-physiology umbrella describing conditions in which intravesical pressure rises (from detrusor contraction or from abdominal straining) during voiding attempts, yet the urethral sphincter fails to relax effectively, resulting in poor emptying. For pragmatic reporting in this study, we used the following operational indicators (reference/warning thresholds rather than diagnostic cut-offs): Pves > 20 cmH_2_O, Qmax < 15 mL/s, and PVR > 50 mL. In our all-male sample, 76/81 (93.83%) met these BSDS criteria, encompassing both classical DSD and the NBOO pattern.

**Figure 3:**
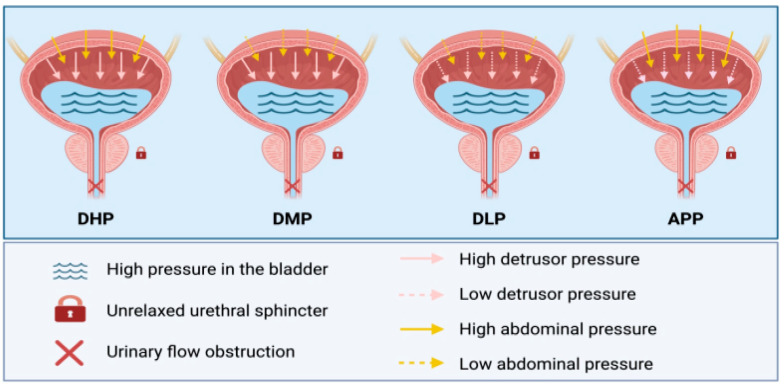
Schematic diagram of the four subtypes of Bladder–Sphincter Dyscoordination Syndrome (BSDS). Clinical takeaway: This conceptual map links each subtype to its dominant pressure source and sphincter condition, illustrating why different subtypes may require different management emphases (e.g., reducing outlet resistance for straining-predominant cases vs. assisting emptying for low-pressure cases).

**Figure 4:**
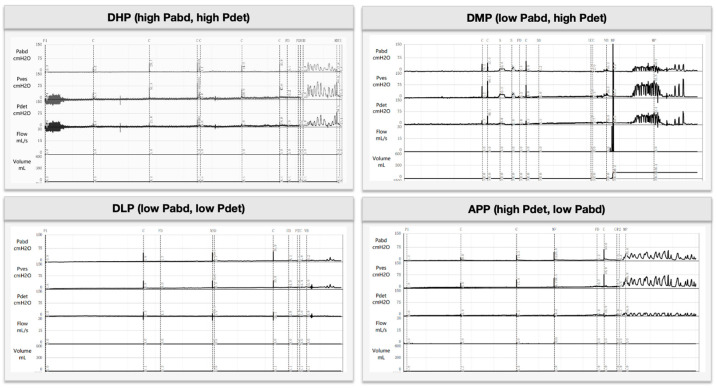
Representative urodynamic pressure–flow traces for each BSDS subtype. Clinical takeaway: Recognizing these distinct pressure patterns (high detrusor vs. high abdominal vs. low pressures) during urodynamics can guide targeted interventions—for example, identifying an Abdominal-Pressure Pattern suggests focusing on sphincter relaxation and avoiding chronic straining.

A conceptual map of the BSDS subtype framework is shown in Figure 3. Representative urodynamic traces for each subtype are provided in Figure 4. To align management with the source of force and outlet resistance, we stratified BSDS into four patterns using reference thresholds (Pdet 20 cmH_2_O; Pabd 40 cmH_2_O):

Dual High-Pressure (DHP): Pdet ≥ 20 cmH_2_O and Pabd ≥ 40 cmH_2_O.

Detrusor-Muscle Predominant (DMP): Pdet ≥ 20 cmH_2_O, Pabd < 40 cmH_2_O (classic DSD aligns with this subtype).

Dual Low-Pressure (DLP): Pdet < 20 cmH_2_O, Pabd < 40 cmH_2_O (compatible with underactive detrusor patterns).

Abdominal-Pressure Pattern (APP): Pdet < 20 cmH_2_O, Pabd ≥ 40 cmH_2_O (voiding largely via straining).

Subtype profiles (Table 4). Descriptively, DMP showed the highest detrusor pressures (e.g., Pdet@CC 33.15 ± 31.82 cmH_2_O) and the lowest compliance (19.23 ± 32.36 mL/cmH_2_O), consistent with a detrusor-driven pattern; APP demonstrated low detrusor pressures with prominent abdominal straining (∆Pabd 72.34 ± 28.31 cmH_2_O) and the poorest emptying (bladder emptying rate 8.49%, PVR 480.44 ± 134.55 mL); and DLP exhibited globally low pressures with high residuals (PVR 494.35 ± 139.60 mL). These profiles suggest distinct mechanistic targets: outlet reduction (e.g., external-sphincter botulinum toxin) for DMP/DHP, and assisted emptying/conditioning for DLP/APP. The subtype thresholds are pragmatic reference levels rather than diagnostic absolutes and are interpreted alongside the full urodynamic trace. Subsequent sections relate each subtype to clinical endpoints and management implications.

## 4. Discussion

Bladder function depends on tightly coordinated activity of the detrusor, bladder neck/internal sphincter region, and the striated external urethral sphincter, orchestrated by supraspinal–spinal circuits and autonomic/somatic efferents [13,14]. In humans, sympathetic outflow to the lower urinary tract arises predominantly from T11–L2 via the hypogastric nerves, parasympathetic outflow from S2–S4 via the pelvic nerves, and somatic sphincter control from Onuf’s nucleus (S2–S4) via the pudendal nerves [13,14]. Accordingly, lesions above the sacral cord primarily disrupt pontine–spinal coordination and can produce detrusor overactivity and detrusor–sphincter dyssynergia (DSD), whereas sacral/infrasacral injuries more often impair the efferent nuclei or peripheral pathways, predisposing to detrusor areflexia/underactivity and inefficient emptying [8,15].

From a clinical standpoint, contemporary NLUTD care is phase-targeted and risk-stratified: storage-phase management prioritizes pressure safety and continence (e.g., antimuscarinics/β3-agonists and, when appropriate, botulinum toxin), whereas voiding-phase failure prioritizes reliable low-pressure emptying—most commonly via clean intermittent catheterization (CIC)—with outlet-directed interventions reserved for selected physiologic patterns [2,8,16]. This “pressure safety first, then emptying optimization” principle is grounded in long-standing evidence that sustained elevated intravesical pressures and poor compliance are associated with upper-tract deterioration in neurogenic bladders [17,18,19,20].

In our LSCI cohort, voiding performance reflected the balance between available driving force (detrusor contraction and, in some patients, compensatory abdominal straining) and outlet resistance (in the absence of fixed anatomic obstruction). DSD, in its classic urodynamic sense, denotes a detrusor contraction occurring concurrently with involuntary contraction of the periurethral/urethral striated sphincter and is classically associated with suprasacral lesions [8,15,20,21,22]. However, many LSCI patients in our cohort exhibited extremely weak detrusor activity, near-zero flow, and reliance on abdominal straining, creating scenarios in which standard pressure–flow metrics that require a definable Pdet@Qmax (e.g., BOOI/BCI) become inapplicable or unreliable—particularly when Qmax approaches zero or when abdominal straining contaminates interpretation [12,23,24]. This practical gap motivated us to adopt an operational descriptive label—neurogenic bladder outlet obstruction (NBOO)—to denote a straining-dependent, poor-emptying phenotype after excluding structural obstruction, characterized in our dataset by high Pabd during voiding attempts with low Pdet and inadequate emptying. Importantly, this usage is not intended to replace established pressure–flow definitions of obstruction; rather, it provides a concise way to communicate a clinically relevant pattern in LSCI in which “flow is mechanically driven” yet ineffective (Table 5).

Mechanistically, abdominal straining does not guarantee efficient antegrade flow. Spinal continence reflexes (“guarding reflex”) and abnormal pelvic-floor activation can increase outlet resistance during attempts to void, effectively counteracting rises in abdominal pressure [13,25]. Thus, in straining-driven voiding, higher Pabd may coexist with low flow if outlet relaxation is delayed, incomplete, or intermittently overridden by reflex guarding.

**BSDS as a pragmatic umbrella for discoordinated voiding attempts in LSCI.** Building on these observations, we propose bladder–sphincter dyscoordination syndrome (BSDS) as a pragmatic clinical–physiology umbrella: during a voiding attempt, intravesical pressure rises (from detrusor contraction and/or abdominal straining), yet effective outlet relaxation is insufficient, resulting in poor emptying. For operational reporting, we stratified BSDS into four pressure-behavior patterns using reference thresholds (Pdet 20 cmH_2_O; Pabd 40 cmH_2_O): Dual High-Pressure (DHP), Detrusor-Muscle Predominant (DMP), Dual Low-Pressure (DLP), and Abdominal-Pressure Pattern (APP). These patterns map onto plausible mechanisms—e.g., DMP is compatible with a detrusor-driven attempt with inferred outlet non-relaxation (DSD-like physiology), whereas APP reflects straining-dominant attempts in the setting of detrusor underactivity.

We emphasize that, without surface EMG or videourodynamics, outlet non-relaxation in our study is inferred from pressure/flow behavior rather than directly visualized; therefore, BSDS is best interpreted as a descriptive framework that organizes clinically observed voiding failures in LSCI rather than a definitive physiologic diagnosis [8].

An important finding is that storage-phase physiology can carry over into voiding-phase performance. Patients with DO exhibited higher filling pressures and, on average, lower residuals and higher emptying rates than non-DO patients. We interpret this cautiously: involuntary detrusor activity originating in the storage phase may transiently augment voiding driving pressure in selected circumstances, yet elevated filling pressures and/or low compliance remain central risk signals that warrant mitigation [2,16,17,19,26]. Thus, DO can simultaneously represent a risk phenotype (leakage, high-pressure exposure) and a potential contributor to emptying in some individuals; clinically, management should prioritize pressure safety and only then optimize emptying strategy [2,16].

**Positioning BSDS/NBOO relative to established ICS terminology.** Traditional ICS frameworks emphasize discrete urodynamic entities such as DSD and DU [8]. DU is defined as a detrusor contraction of reduced strength and/or duration, resulting in prolonged or incomplete emptying [27]. In our taxonomy, the DLP subtype captures a DU-like voiding failure pattern within a discoordination framework—low pressures and poor emptying—highlighting that, in LSCI, underactive detrusor function may co-present with functionally increased outlet resistance or reflex guarding, even without classic high-pressure DSD signatures. Similarly, NBOO corresponds to a form of functional outlet obstruction driven by delayed/inadequate relaxation during straining-dominant voiding attempts. Analogous “delayed sphincter relaxation” pressure–flow patterns have been described in other neurologic conditions, including Parkinson’s disease [28], supporting the plausibility of such a mechanism even though the neuroanatomical substrate differs from LSCI.

Conceptually, BSDS is intended to unify common LSCI voiding-failure presentations (detrusor-driven high-pressure attempts, low-pressure DU-like failure, and straining-dominant attempts) under a single descriptive umbrella to facilitate structured clinical communication and hypothesis generation regarding treatment priorities [2,8].

**Linking subtypes to management.** Our subtype-to-management mapping should be interpreted as physiologically grounded hypotheses. APP (low Pdet, high Pabd) represents the clearest scenario where outlet-directed approaches (e.g., urethral/external sphincter botulinum toxin) may reduce functional outlet resistance and potentially lower the effort required for voiding—recognizing the tradeoff of stress incontinence and the need for individualized dosing and counseling [2,29,30,31,32,33,34]. DLP (low Pdet, low Pabd) emphasizes assisted emptying (CIC) and bladder protection, as outlet reduction alone may not overcome insufficient detrusor force [2,27]. DMP/DHP patterns require careful separation of volitional voiding attempts from involuntary high-pressure storage carry-over; in either case, storage pressure control should precede more aggressive outlet lowering [2,18].

**Safety considerations.** Two practical safety issues warrant emphasis. First, post-sphincter chemodenervation incontinence is a predictable risk of urethral sphincter botulinum toxin; dosing should be individualized to baseline continence status and urodynamic context [29,31,34]. Second, chronic reliance on Credé/Valsalva is generally discouraged as a primary long-term emptying strategy because it can be associated with high intravesical pressures and urologic complications; long-term observational data and reviews in SCI populations caution against its routine use [35,36,37]. When straining is used transiently, it should be guided by urodynamics, with clear stop thresholds and continued upper-tract surveillance.

**Digital monitoring and the limits of flow-only assessment in LSCI.** Telemedicine and home monitoring for LUTS are expanding. A low-cost home uroflowmetry system enabling telemonitoring has been reported and exemplifies the growing emphasis on objective flow data outside the clinic [38]. However, in LSCI, flow may be weak or “mechanically” generated by abdominal pressure in the setting of detrusor hypocontractility, so flow rate alone may not adequately characterize voiding physiology. A pressure-behavior framework such as BSDS may therefore complement flow-centered assessments by explicitly incorporating the relative contributions of detrusor and abdominal pressure alongside inferred outlet resistance.

**Generalizability.** Our cohort was male-predominant, and male outlet anatomy (including prostatic factors, even when overt obstruction is excluded) differs fundamentally from the female outlet. Female bladder outlet obstruction is increasingly recognized as underdiagnosed and heterogeneous, with functional sphincteric obstruction representing a substantial subset in specialized centers [39,40,41]. Therefore, the prevalence of patterns such as NBOO/APP and the optimal pressure thresholds may differ in women with LSCI. Dedicated validation in female cohorts is needed before extrapolating BSDS thresholds or subtype frequencies.

**Limitations and future work.** This retrospective, descriptive study cannot establish causality or prognostic performance. Sample size was modest, concurrent upper-tract imaging was incomplete, and the chosen pressure thresholds should be viewed as pragmatic reference points rather than biologic cutoffs. Most importantly, we lacked surface EMG and video-urodynamics, so outlet behavior was inferred from pressure/flow traces, which may misclassify some cases. Prospective studies incorporating full neuro-urodynamic assessment and linking BSDS subtypes to clinically meaningful outcomes (upper-tract safety, continence, catheter dependence, infections, and treatment response) are needed to test whether BSDS is predictive and actionable.

In summary, our analysis proposes BSDS (with its subtypes) and the operational NBOO label as descriptive tools to organize voiding dysfunction after LSCI, emphasizing pressure safety and the mechanism of attempted emptying. These constructs are hypothesis-generating and require prospective validation before routine adoption.

## 5. Conclusions

In men with LSCI, urodynamic evaluation commonly reveals some form of bladder–sphincter discoordination affecting voiding. We identified four patterns within a proposed BSDS framework (DHP, DMP, DLP, APP) and defined a descriptive NBOO category for cases where voiding relies on straining with minimal detrusor effort. These study-specific constructs are intended as readable descriptors that link the source of voiding force to outlet behavior—emphasizing safety (avoiding high pressures) before completeness of emptying. Our findings suggest that such a classification is feasible and may have clinical relevance, but we stress that it does not replace established pressure-flow diagnostic criteria. Rather, it provides an additional perspective in scenarios where standard indices (like BOOI/BCI) are inapplicable (e.g., when flow is near zero or voiding is by Valsalva). Future directions: Prospective validation of the BSDS scheme is warranted. In particular, correlating each subtype with outcomes—upper tract status, continence, need for catheterization—will be crucial to determine its true utility. Until then, the concept should be viewed as hypothesis-generating. By laying this groundwork, we hope to stimulate further research and ultimately improve individualized management of neurogenic voiding dysfunction after LSCI.

## Figures and Tables

**Table 1:** Baseline characteristics of patients meeting BSDS criteria, stratified by subtype.

	Total (*n* = 76)	DHP (*n* = 22)	DMP (*n* = 13)	DLP (*n* = 15)	APP (*n* = 26)	*p*-Value
Age, years	41.01 ± 14.40	40.86 ± 14.49	39.15 ± 11.14	38.13 ± 13.00	43.73 ± 16.64	0.711
Time, months	7.95 ± 8.07	8.91 ± 8.68	7.23 ± 4.83	7.30 ± 7.36	7.88 ± 9.44	0.828
Injury type						0.146
–Spinal cord injury	39 (51.3%)	11 (50.0%)	10 (76.9%)	5 (33.3%)	13 (50.0%)	
–Cauda equina syndrome	37 (48.7%)	11 (50.0%)	3 (23.1%)	10 (66.7%)	13 (50.0%)	
Urologic history						—
–Any prior urologic disease	0 (0%)					
–None	76 (100%)					
Bladder management						0.167
–CIC (clean intermittent catheterization)	64 (84.2%)	22 (100%)	9 (69.2%)	12 (80.0%)	21 (80.8%)	
–IC (indwelling catheter)	10 (13.2%)	0	3 (23.1%)	2 (13.3%)	5 (19.2%)	
–Non-catheterized	2 (2.6%)	0	1 (7.7%)	1 (6.7%)	0	

**Table 2:** Comparison of urodynamic performance with and without involuntary contraction of detrusor muscle.

	Total (81)	DO (33)	Non-DO (48)	*p*-Value
Age (years)	40.43 ± 14.18	39.36 ± 13.97	41.17 ± 14.42	0.577
Time (months)	7.56 ± 6.27	8.76 ± 6.61	6.73 ± 5.95	0.067
Capacity@FD (mL)	239.19 ± 126.33	204.37 ± 127.42	265.30 ± 120.40	**0.035**
Pves@FD (cmH_2_O)	16.12 ± 17.08	25.22 ± 22.64	9.29 ± 4.78	**<0.001**
Pdet@FD (cmH_2_O)	11.05 ± 16.30	19.13 ± 22.28	4.99 ± 3.47	**0.001**
CC (mL)	478.31 ± 110.12	451.33 ± 120.38	496.86 ± 99.56	0.105
Pves@CC (cmH_2_O)	28.57 ± 23.65	45.36 ± 28.41	17.03 ± 8.25	**<0.001**
Pdet@CC (cmH_2_O)	19.64 ± 19.46	34.28 ± 22.80	9.58 ± 6.04	**<0.001**
Compliance (mL/cmH_2_O)	33.68 ± 29.88	12.58 ± 8.30	53.46 ± 29.28	**<0.001**
Qmax (mL/s)	3.52 ± 7.18	5.16 ± 9.10	2.39 ± 5.30	**0.031**
Pves@Qmax (cmH_2_O)	54.75 ± 35.50	54.99 ± 27.68	54.47 ± 43.75	0.375
Pdet@Qmax (cmH_2_O)	28.07 ± 19.55	34.19 ± 16.84	20.64 ± 20.60	**0.006**
Pves@VB (cmH_2_O)	31.02 ± 25.72	46.21 ± 30.15	20.35 ± 14.91	**<0.001**
Pdet_max (cmH_2_O)	42.11 ± 32.95	58.53 ± 27.17	30.82 ± 32.03	**<0.001**
ΔPdet (cmH_2_O)	22.47 ± 28.91	24.25 ± 24.43	21.24 ± 31.81	0.188
ΔPabd (cmH_2_O)	62.48 ± 43.90	69.26 ± 43.94	57.61 ± 43.69	0.148
PVR (mL)	421.18 ± 170.29	359.84 ± 163.38	464.25 ± 163.22	**<0.001**
bladder emptying rate (%)	15.66 ± 26.84	23.13 ± 30.24	10.73 ± 23.39	**0.021**
MUP (cmH_2_O)	126.06 ± 47.17	131.80 ± 54.08	122.18 ± 42.08	0.384
MUCP (cmH_2_O)	116.67 ± 45.01	124.11 ± 54.20	111.65 ± 37.40	0.271

**Note**: Capacity@FD: Bladder volume at first desire to void; Pves@FD: Intravesical pressures at first desire to void; Pdet@FD: Detrusor pressure at first desire to void; CC: Cystometric Capacity, Bladder volume at the end of filling cystometry; Pves@CC: Intravesical pressures at the end of filling cystometry; Pdet@CC: Detrusor pressure at the end of filling cystometry; Compliance: Divide the change in volume by the simultaneous change in detrusor pressure during filling cystometry. Less than 20 mL/cmH_2_O indicates a decrease in compliance, representing a decrease in bladder holding capacity; Qmax: maximum urinary flow rate; Pves@Qmax: Intravesical pressures at maximum urinary flow rate; Pdet@Qmax: detrusor pressure at maximum urinary flow rate; Pves@VB: Intravesical pressures at the beginning of voiding; Pdet_max: Maximum detrusor pressure at the voiding stage; ΔPdet: Change in detrusor pressure, ΔPdet = Pdet_max − Pdet@CC; ΔPabd: Maximum change in abdominal pressure, ΔPabd = Pabd@max − Pabd@CC; PVR: Post-Void Residual of urine volume; bladder emptying rate = (voided volume/CC) × 100%; MUP: Maximum Urethral Pressure; MUCP: Maximum Urethral Closure Pressure. Values are mean ± SD unless stated; group tests as per Section 2.6.

**Table 3:** Comparison of Detrusor-Sphincter Dyssynergia (DSD) and Neurogenic Bladder with Outlet Obstruction (NBOO).

	DSD (31)	NBOO (32)	*p* Value
Age (years)	41.35 ± 13.14	41.50 ± 16.11	0.220
Time (months)	8.90 ± 7.48	6.69 ± 4.84	0.351
Capacity@FD (mL)	202.23 ± 124.81	273.77 ± 135.19	**0.0** **25**
Pves@FD (cmH_2_O)	21.49 ± 19.69	14.84 ± 16.93	0.083
Pdet@FD (cmH_2_O)	16.78 ± 20.42	8.77 ± 14.35	**0.0** **33**
CC (mL)	456.26 ± 129.75	491.27 ± 96.01	0.514
Pves@CC (cmH_2_O)	39.39 ± 29.02	24.01 ± 18.83	**0.0** **17**
Pdet@CC (cmH_2_O)	29.02 ± 24.39	16.23 ± 14.99	**0.0** **27**
Compliance (mL/cmH_2_O)	22.97 ± 31.02	38.77 ± 30.65	**0.00** **9**
Qmax (mL/s)	5.29 ± 9.43	1.46 ± 2.77	0.083
Pves@Qmax (cmH_2_O)	64.77 ± 40.72	48.76 ± 34.21	0.223
Pdet@Qmax (cmH_2_O)	33.49 ± 18.49	21.56 ± 20.39	**0.040**
Pves@VB (cmH_2_O)	44.78 ± 31.89	24.77 ± 16.43	**0.009**
Pdet_max (cmH_2_O)	64.91 ± 33.39	32.14 ± 22.82	**<0.001**
ΔPdet (cmH_2_O)	35.89 ± 35.68	15.91 ± 15.87	**0.005**
ΔPabd (cmH_2_O)	61.47 ± 43.45	81.51 ± 33.38	**0.019**
PVR (mL)	363.69 ± 167.65	475.76 ± 128.12	**0.004**
bladder emptying rate (%)	20.21 ± 28.86	8.46 ± 15.13	**0.029**
MUP (cmH_2_O)	125.65 ± 53.79	125.06 ± 44.56	0.413
MUCP (cmH_2_O)	116.51 ± 53.53	113.92 ± 39.17	0.502

**Table 4:** Urodynamic characteristics of Bladder-Sphincter Dyscoordination Syndrome and its subtypes.

	BSDS (76)	DHP (22)	DMP (13)	DLP (15)	APP (26)
Age (years)	41.01 ± 14.40	40.86 ± 14.49	39.15 ± 11.14	38.13 ± 13.00	43.73 ± 16.64
Time (months)	7.45 ± 6.10	8.86 ± 7.90	7.00 ± 3.58	6.93 ± 6.47	6.77 ± 5.22
Capacity@FD (mL)	245.63 ± 127.37	201.72 ± 127.43	205.26 ± 117.51	265.58 ± 81.74	293.12 ± 138.13
Pves@FD (cmH_2_O)	16.43 ± 17.32	15.27 ± 13.43	25.24 ± 25.05	9.39 ± 5.52	16.48 ± 18.39
Pdet@FD (cmH_2_O)	11.21 ± 16.64	10.91 ± 11.98	20.56 ± 27.46	4.75 ± 2.25	9.97 ± 15.77
CC (mL)	480.67 ± 109.18	454.12 ± 116.79	449.51 ± 138.67	519.21 ± 71.71	496.49 ± 99.58
Pves@CC (cmH_2_O)	29.14 ± 23.91	32.80 ± 26.78	40.95 ± 31.59	20.39 ± 10.95	25.18 ± 20.50
Pdet@CC (cmH_2_O)	19.99 ± 19.83	21.60 ± 17.55	33.15 ± 31.82	10.41 ± 5.80	17.57 ± 16.22
Compliance (mL/cmH_2_O)	34.07 ± 30.82	29.66 ± 29.86	19.23 ± 32.36	47.56 ± 19.36	39.31 ± 33.63
Qmax (mL/s)	2.20 ± 3.94	3.08 ± 5.06	3.42 ± 4.79	1.35 ± 3.40	1.35 ± 2.24
Pves@Qmax (cmH_2_O)	54.53 ± 38.45	95.91 ± 42.91	34.83 ± 18.77	30.67 ± 5.28	40.39 ± 21.06
Pdet@Qmax (cmH_2_O)	26.86 ± 19.80	36.68 ± 24.79	24.67 ± 11.84	15.73 ± 5.14	23.31 ± 20.81
Pves@VB (cmH_2_O)	30.34 ± 25.87	41.31 ± 33.11	43.62 ± 18.77	14.46 ± 7.80	23.99 ± 14.72
Pdet_max (cmH_2_O)	41.59 ± 32.84	62.34 ± 35.39	62.30 ± 29.36	14.27 ± 7.02	29.45 ± 23.82
ΔPdet (cmH_2_O)	21.60 ± 28.08	40.73 ± 37.83	29.15 ± 25.07	3.86 ± 9.19	11.87 ± 13.23
ΔPabd (cmH_2_O)	60.71 ± 41.99	96.99 ± 39.32	25.54 ± 12.18	19.39 ± 12.37	72.34 ± 28.31
PVR (mL)	442.13 ± 152.84	414.48 ± 148.33	344.53 ± 174.45	494.35 ± 139.60	480.44 ± 134.55
bladder emptying rate (%)	11.36 ± 21.82	12.82 ± 23.53	20.37 ± 30.42	10.12 ± 22.71	8.49 ± 14.16
MUP (cmH_2_O)	124.95 ± 46.49	129.49 ± 54.45	113.80 ± 49.56	129.67 ± 42.32	124.28 ± 42.02
MUCP (cmH_2_O)	115.18 ± 44.13	114.60 ± 52.60	106.62 ± 45.98	122.22 ± 41.05	115.86 ± 39.23

**Table 5:** Conceptual comparison of BSDS/NBOO classification vs. traditional urodynamic terms.

BSDS/NBOO Term	Comparable Classical Term (s)	Distinction/Clarification
Bladder–Sphincter Dyscoordination Syndrome (BSDS)	Detrusor–Sphincter Dyssynergia (DSD) (ICS definition)	Broader concept: BSDS encompasses any voiding attempt where intravesical pressure rises (from detrusor contraction or straining) without effective sphincter relaxation. It includes classical DSD but also cases with minimal detrusor activity. In contrast, ICS-defined DSD specifically refers to involuntary sphincter contraction during a detrusor contraction (typically in suprasacral SCI). BSDS extends this to include lower-level injuries where detrusor contraction may be weak or absent.
Neurogenic Bladder Outlet Obstruction (NBOO)	Functional outlet obstruction with detrusor underactivity (no exact single term in ICS)	Descriptive label: NBOO denotes straining-dependent voiding failure in LSCI—i.e., the patient must Valsalva to void, and emptying is incomplete despite no anatomical block. Traditionally, such cases might be classified as “detrusor underactivity with functional obstruction (non-relaxing sphincter)” or simply underactive bladder if the outlet actually relaxes. NBOO encapsulates both scenarios since we infer outlet dysfunction when straining is insufficient. It is not an official ICS term, but highlights a common LSCI phenotype where the bladder contraction is negligible and voiding is hampered by outlet resistance (e.g., incomplete sphincter relaxation).
DMP (Detrusor-Muscle Predominant)	Classic DSD (Type III according to ICS neurogenic classification)	High detrusor pressure, low abdominal effort: DMP cases have a strong detrusor contraction with little need for abdominal strain, yet voiding is incomplete. This pattern aligns with classical DSD—a contracting detrusor opposed by a non-relaxing sphincter. In other words, DMP is how DSD presents in our framework (often seen in suprasacral injuries).
APP (Abdominal-Pressure Pattern)	Straining (Valsalva) voiding without true obstruction (vs. functional obstruction)	High abdominal pressure, low detrusor pressure: APP describes voiding largely driven by abdominal strain (Pabd ≥ 40) with an underactive detrusor (Pdet < 20). If the sphincter is actually relaxing normally, APP corresponds to an extreme form of detrusor underactivity compensated by Valsalva (no genuine obstruction). If the sphincter is not relaxing, APP overlaps with the functional obstruction concept (effectively our NBOO). Without EMG or videourodynamics, APP cases in our study are labeled NBOO by assumption. The key difference from classic obstruction: there is no mechanical blockage, only lack of coordinated detrusor–sphincter function.
DLP (Dual Low-Pressure)	Detrusor underactivity (underactive bladder, acontractile detrusor)	Low detrusor, low abdominal pressures: DLP represents a feeble or absent detrusor contraction with no significant straining compensation. This mirrors a pure detrusor underactivity scenario in classical terms. Patients cannot generate meaningful pressure to void (hence very low flow, high residual), corresponding to what is traditionally managed as acontractile or severely underactive bladder.

## Data Availability

The original contributions presented in the study are included in the article. Further inquiries can be directed to the corresponding author.

## Abbreviations

The following abbreviations are used in this manuscript:

LSCI	Lower-level spinal cord injury
NLUTD	Neurogenic lower urinary tract dysfunction
BSDS	Bladder–sphincter dyscoordination syndrome
DO	Detrusor overactivity
CIC	Clean intermittent catheterization
DU	Detrusor underactivity
DSD	Detrusor–sphincter dyssynergia
BOOI	Bladder outlet obstruction index
DHP	Dual High-Pressure
DMP	Detrusor-Muscle Predominant
DLP	Dual Low-Pressure
APP	Abdominal-Pressure Pattern
